# Religiosity and Fertility Intentions: Can the Gender Regime Explain Cross-Country Differences?

**DOI:** 10.1007/s10680-020-09574-w

**Published:** 2021-02-05

**Authors:** Christoph Bein, Anne H. Gauthier, Monika Mynarska

**Affiliations:** 1 Netherlands Interdisciplinary Demographic Institute (NIDI-KNAW), The Hague, The Netherlands; 2 University of Groningen, Groningen, The Netherlands; 3 Cardinal Stefan Wyszyński University in Warsaw, Warsaw, Poland

**Keywords:** Religiosity, Fertility intentions, Gender, Cross-national

## Abstract

Research on the relationship between religiosity and fertility intentions revealed substantial cross-national differences. In some countries, a strong and positive effect of religiosity on fertility intentions was found, while in others, the effect was weaker or not significant, and the reasons underlying these cross-national differences are still unclear. The aim of this article is to explain these macro-level differences from the perspective of the prevailing gender regime. We argue that in countries with more traditional regimes, a stronger effect of religiosity on fertility intentions could be expected than in countries with a more egalitarian view. We make use of the first wave of the Generations and Gender Survey and incorporate data from a total of 12 European countries in our analysis. We examine the influence of gender regime according to various macro-level indicators on gender attitudes and gender equality using meta-regression analyses. We also conduct robustness checks using other indicators such as the Gender Development Index. Our results reveal that the gender regime is only able to explain these differences in certain situations, specifically those relating to the long-term fertility intentions of men.

## Introduction

Research on the impact of religiosity on fertility is a re-emerging field in demography and coincides with a general renewed interest in religion in the public discourse (Hubert 2015). In the past, the focus was mainly on the examination of differences in fertility behaviour among different religious denominations, such as Catholics, Protestants and Jews (van Poppel 1985; Westoff and Jones 1979) or Muslims in Europe (Kaufmann et al. 2011). Recently, however, there has been a trend in favour of studies on the influence of individual religiosity on fertility. This is partly related to the increasing secularization which has led to a greater heterogeneity of religious practices among people within each denomination (Kaufmann 2010). Results from such studies show that religiosity tends to be a better predictor of fertility than simple denominational affiliation alone (Adserà 2006a). In general, more religious respondents tend to have more children and have a higher ideal number of children than less religious respondents (Adserà 2006a, b).

The positive relationship between religiosity and fertility behaviour was confirmed using various indicators for religiosity and in a range of geographical settings across Christian-dominated Western countries (Berghammer 2012; Westoff and Frejka 2007; Frejka and Westoff 2007; Hubert 2015; Peri-Rotem 2016). Furthermore, some studies found similar results for the relationship between religiosity and predecessors of fertility behaviour, such as fertility ideals or intentions (Dilmaghani 2019; Hayford and Morgan 2008; Philipov and Berghammer 2007). But this relationship cannot be said to be universal across Western countries. Philipov and Berghammer (2007) revealed considerable differences across countries in terms of the impact of religiosity on fertility ideals, intentions and behaviours. In many countries, the frequency of attending religious services is positively associated with the intention to have a child, but in some countries, this is not the case.

The reasons for such cross-national differences remain somewhat unclear. While Philipov and Berghammer (2007) suggested that religiosity might interact with other social systems relevant to fertility, such as education, work or culture, they did not actually test this hypothesis. In another cross-national study, but restricted to reproductive behaviour, Guetto et al. (2015) concluded that religiosity exerts a stronger effect on the number of children women have in more traditional countries as compared to more secular and liberal contexts. In this paper, we build on the argument that the cultural or institutional setting of a country may moderate the individual-level relationship between religiosity and fertility intentions. We do so by focusing on the gender regime. As discussed later, this allows us to link the gendered nature of religions, and their views on gender roles (at the macro-level), with religiosity (at the micro-level). It also allows us to operationalise differences between more traditional and liberal contexts along both attitudinal or normative, and institutional dimensions. Our hypothesis is that the countries’ gender regime is modifying the individual-level relationship between religiosity and fertility intentions and is consequently key to understanding cross-national differences in this individual-level relationship.

In this paper, this hypothesis is examined by using data from 12 European countries from the Generations and Gender Survey (GGS). Because of the small number of countries, we apply logistic meta-regression. Models are constructed separately according to gender and categorized into short-term (over the next three years) and long-term (over an indefinite time span) intentions. Our results show that in some countries, there is no significant impact of religiosity on fertility intentions, while in other countries, there is a strong positive impact. The gender regime explains only part of these differences, however, as we find evidence for the influence of the attitudinal dimension of gender regime on the effect of religiosity on fertility intentions for men only.

## Theoretical Background and Hypotheses

In the literature, childbearing is often modelled as an outcome of a holistic decision-making process from ideals/desires, through intentions, to behaviour (Ajzen and Klobas 2013; Bachrach and Morgan 2013; Johnson-Hanks et al. 2011; Mencarini et al. 2015; Miller 1994, 2011; Mynarska and Rytel 2018). Fertility ideals and desires stand at the beginning of this process and describe the most basic wishes related to childbearing. While both terms are often used interchangeably (Hin et al. 2011), it has been argued that “desires” may be understood by respondents in terms of personal wishes, while “ideals” may also refer to societal ideas or norms (Knodel and Prachuabmoh 1973; Philipov and Bernardi 2011). Those ideals and desires form the base for fertility intentions, which are the focus of our study. They refer to a concrete plan to have a child or not within a set timeframe. Compared to ideals and desires, intentions take into account restrains that could stand in the way of realizing wishes, such as the opinion of significant others, employment/unemployment, partnership status or the financial status (Iacovou and Tavares 2011; Miller 1994). Intentions are therefore usually a good predictor of actual behaviour (e.g. Régnier-Loilier and Vignoli 2011).

Religiosity—the second concept central for our study—is similarly complex. In general, it refers to the strength of commitment to a religion or religious beliefs, together with the degree of participation in religious activities (Lehrer 2004). Various ways of capturing religiosity have been used in research on the relationship between religiosity and fertility, such as the importance of god or religion in one’s life (Zhang 2008), or by self-assessment of religiosity (Philipov and Berghammer 2007). Furthermore, many researchers used the frequency of attendance at religious services (Berghammer 2012; Dilmaghani 2019; Hubert 2015; Peri-Rotem 2016), believing that it is a more objective indicator of religiosity. From a theoretical point of view, frequency of attendance also expresses the extent of exposure to other religious people and religious doctrines.

### Individual-Level Impact of Religiosity on Fertility Intentions

Religiosity can influence the childbearing decision-making process in various ways. In particular, pronatalist religious doctrines are central to an understanding of this impact. C. Goldscheider (1971) summarized the influence of direct religious doctrines under the term “particularized theology” and stressed that many religions explicitly encourage their followers to have large number of children. Furthermore, religions often emphasize family values associated with parenthood, such as the importance of marriage and the role of women as mothers and carers (McQuillan 2004). The Catholic Church, for example, has developed a set of defined pronatalist rules and values concerning marriage and family (Catechism 2366–2379). A high level of religiosity also leads to more contact and stronger relationships with other highly religious people, who may harbour similar opinions on family and children (Krause et al. 2001; Peri-Rotem 2016; Philipov and Berghammer 2007). In other words, one way religiosity can influence the childbearing decision-making process is through fertility ideals, that is, the promotion of a larger family size.

Another path of influence concerns the influence of religiosity on actual fertility behaviour. This manifests itself, for example, in the stance of religions on the use of contraception. While Protestant and Orthodox denominations are less critical of the use of contraception (Hubert 2015), the Catholic Church has very strict rules regarding its use (Srikanthan and Reid 2008), which could also influence the reproductive choices of its followers. Furthermore, religious doctrines may also have a negative influence on fertility behaviour, by inhibiting extramarital births, for example, among teenagers (Lyons and Smith 2014) or delaying the entrance into sexual activity (Manlove et al. 2008).

When it, however, comes to fertility intentions, additional mechanisms may be at work. In particular, and as noted by Philipov (2011), religious people could form fertility intentions differently due to their belief in a higher power that would support them in raising children and save them from the worries and concerns of parenthood. There is already some empirical evidence that for the more religious, perceived costs regarding childbearing matter less in their childbearing decisions compared to the less religious (Arránz Becker and Lois 2017; Bein et al. 2020). This could imply that the more religious may formulate fertility intentions more frequently than the less religious, as constraints that inhibit the translation of fertility ideals into intentions play a less prominent role among them. Notably, attitudes towards marriage or contraceptives, shaped by religion, might also have an impact on childbearing intentions.

Overall, the exposure to religious doctrines and contacts with highly religious groups may have a direct or an indirect impact on all stages of the reproductive decision-making process: from ideals to actual behaviours. In our study, we focus on the impact of religiosity on intentions, constituting the middle part of the decision-making process. We examine the role of religiosity for both short- and long-term childbearing intentions. Short-term fertility intentions describe whether a respondent plans to have a child, or another child, within a fixed (short) period (in our case, over the next three years). Long-term intentions refer to a plan to have a child, or another child, at any point in the future. Short-term intentions thus capture both the timing and quantum of fertility, while long-term intentions capture the quantum only. Therefore, long-term intentions are closer to concepts such as “total intended (or expected) family size” and also closer to fertility ideals (Philipov and Bernardi 2011). These indicators relating to the quantum of fertility have been proven to be decisively affected by religiosity (Hayford and Morgan 2008; Philipov and Berghammer 2007). Evidence for religious influence on the timing element, which constitutes an important part of the short-term intentions, is less clear (Hayford and Morgan 2008). These considerations at the micro-level lead to the following two hypotheses:

#### H1a

*Religiosity is expected to be positively related to fertility intentions (i.e., the more religious a respondent, the more likely he/she is to intend to have a child, or another child).*

#### H1b

*Religiosity is expected to have a stronger effect on long-term than short-term fertility intentions.*

### Macro-level Moderating Impact of the Gender Regime on the Association Between Religiosity and Fertility Intentions

Previous research found considerable variation in the relationship between religiosity and various indicators of fertility across countries, including a rather strong and clear impact of religiosity on fertility ideals, intentions and behaviour in some countries, and no effect at all in others (Adserà 2006b; Hubert 2015; Peri-Rotem 2016; Philipov and Berghammer 2007). Some researchers, like Adserà (2006b), suggested that the religious context in each country plays a role in determining to what extent fertility ideals differ between the more and the less religious. Okun (2017) also stressed the role of religious context and noted that for Jews in Israel, religious community and the role of religion as an institutional power can explain individual differences in the relationship between religiosity and fertility. In contrast, other authors argued that the micro-level relationship between religiosity and fertility might be influenced by other contextual variables. For instance, Guetto et al. (2015) considered the overall role of culture and noted that religiosity has a stronger impact on fertility in more traditional and Catholic countries. And Philipov and Berghammer (2007) assumed that individual-level religiosity might interact with other social systems in shaping fertility. Overall, any interdependencies in people’s life course are strongly dependent on different cultural and institutional contexts in which they occur (Bernardi et al. 2019). These contexts need to be considered when analysing relationship between childbearing intentions and religiosity.

In our study, we refer to gender regimes to capture the cultural (normative, attitudinal) and institutional context in which childbearing takes place. The concept of gender regime was initially developed as an alternative to Esping-Andersen’s welfare state classification (Betzelt 2007). Consequently, the first gender-regime typologies focus mainly on the institutional part of gender equality and examined the extent to which women are enabled by institutions and policies to have access to the labour market and to lead a household independently (e.g. Lewis 1992; Sainsbury 1999; Walby 2004). These typologies were usually conceived as a spectrum of regimes, ranging from those supporting the male-breadwinner (traditional) model all the way to those supporting the individual-adult or egalitarian model. Later, the definition of gender regime was widened to take account of the cultural-normative dimension of gender equality (Betzelt 2007; MacRae 2006). That way, the gender regime can capture the contrast that has often been made in demographic studies between the familialist and traditional cultures of Southern Europe and the individualistic Northern European countries where gender equality is emphasized (e.g. Dalla Zuanna 2001; Livi-Bacci 2001).

The mechanism by which the gender regime may influence the individual-level relationship between religiosity and fertility intentions is centred around the fact that religions themselves are gendered institutions (Neitz 2014). Many religions propagate traditional gender roles and have different rules in place for men and women. For example, in the Catholic Church or the Russian Orthodox Church only men are allowed to become priests (Chernyak 2016; Haskins 2003). Most religions have also created mechanisms regarding gender relationships in the private sphere, emphasizing the ideal of the woman as a caring mother and a good wife. Men, on the other hand, are expected to be good husbands and providers. Many religions are critical of gender equality or the emancipation of women (Klingorová and Havlicek 2015). C. Goldscheider (2006) argued that the promotion of these traditional gender roles plays a role in the influence of religiosity on fertility, even though the direct pronatalist teaching may also be influential (Bein et al. 2017).

The hypothesized mechanism of how the gender regime moderates a micro-level relationship between religiosity and fertility could thus involve the extent to which the gender roles supported by the gender regimes (at the macro-level) are in line with those expressed at the individual level. For example, traditional gender regimes generally emphasize male-breadwinner family models, just as most religions do. In such a country context, the preferred gender arrangement of more religious individuals is mostly in line with the ruling gender regime in society, meaning that religious people experience institutional and societal support for the choices they make. This compatibility between the macro- and micro-sphere is consequently expected to modify the individual-level relationship between religiosity and fertility. Specifically, we therefore expect the strength of the relationship to be larger in traditional gender regimes. Concretely, what this means is that the relationship between religiosity and fertility intentions would be expected to be stronger in a country such as Poland where a support for more traditional gender roles still prevails, and weaker in a country such as Sweden where a support for more gender equal roles is in place. Our second hypothesis thus is as follows:

#### H2

*The effect of religiosity on fertility intentions is stronger in countries with a more traditional gender regime.*

In this study, we consider men and women separately. This is especially crucial in our case, as gender regimes may impact men’s and women’s lives differently. Additionally, most studies on the relationship between religiosity and fertility focused exclusively on the situation of women. Of those studies that considered both men and women, Zhang (2008) did not find any differences in that relationship between men and women for the USA. In contrast, a study by Hubert (2015) on the situation in France, Hungary, Norway and Germany concluded that religiosity is a stronger determinant of male fertility. Our study will generate new evidence on the differences between men and women.

## Data and Methods

### Dataset and Sample Selection

Our data source is Wave 1 of the Generations and Gender Survey (GGS). For the purpose of this study, we selected those twelve countries for which the GGS provides data on frequency of attendance at religious services and both long- and short-term fertility intentions^1^: of these, four countries are dominated by Orthodox Christianity (Bulgaria, Russia, Georgia and Romania), four are majority Catholic (France, Austria, Lithuania and Poland), two are majority Protestant (Norway and Sweden), Czechia is majority unaffiliated and Germany is mixed Catholic and Protestant. The data were collected between 2004 and 2013. Our sample was restricted to men (18–49 years) and women (18–44 years) of childbearing age (*N* = 72,071). We further restricted our sample by omitting cases for which the survey questions on fertility intentions were not deemed applicable, such as for those indicating infertility, of themselves or their partners (Beaujouan 2013), or declared themselves pregnant, resulting in 66,074 respondents. Missing values on fertility intentions and religiosity further reduced the sample to *N* = 54,429 respondents, of which there were 25,832 men and 28,597 women.

### Measurement of Religiosity

Following many previous studies (Berghammer 2012; Dilmaghani 2019; Hubert 2015; Peri-Rotem 2016), the paper uses frequency of attendance at religious services as a marker for religiosity. In the GGS, attendance frequency was asked with the following question: “How often, if at all, do you attend religious services (apart from weddings, funerals, baptisms, and the like)?” In most countries, respondents were able to give numerical responses, such as “10 times a year” or “2 times a week”. In others, religiosity was assessed via a varying set of categories, such as “more than once per month” or “once per year”. In order to harmonize the variable across countries, we recoded attendance frequency into three classes: “never” (also called “low religiosity” in our models), “less than monthly” (medium religiosity) and “monthly or more often” (high religiosity).

### Other Micro-Level Variables

The dependent variable is a measure of fertility intentions, in other words, whether the respondent intends to have a(nother) child. In the GGS, fertility intentions were assessed using two questions. The first concerns fertility intentions over the next three years (short term) and was asked as follows: “Do you intend to have a(nother) child over the next three years?”. Respondents who answered that they did not intend to have a(nother) child during that time were asked an additional question on their intentions beyond that time frame: “Supposing you do not have a/another child during the next three years, do you intend to have any (more) children at all?” For both questions, respondents were able to answer on a four-point scale ranging from “definitely yes” to “definitely no” in most countries. In Norway, however, only a binary answer was possible (yes or no). For this reason, we recoded all answers into a binary format (1: intends to have a child, 0: does not intend to have a child).

In order to create a variable that captures fertility intentions irrespective of the time frame (which we now refer to as “long-term intentions”), we combined the answers to the two aforementioned questions in the following way. Those respondents who answered yes either for the short-term intentions or for their intentions beyond three years were coded as “yes”, while those respondents who answered no to both questions were coded as “no”.

By measuring religiosity in terms of frequency of attendance as described in Sect. 3.2, its meaning could vary depending on the religion concerned. We therefore included religious affiliation as a control variable. Unfortunately, for Germany, detailed information on different Christian denominations was missing. Additionally, there are too few cases to permit separate categories to be used for non-Christian religions such as Islam or Judaism. With these limitations in mind, we divided religious affiliation into three groups (Christian, other religion, unaffiliated). We also included the following additional control variables: age of respondent, number of biological children, whether the respondent had a child younger than 10 years old, partnership status, education level (based on ISCED categories: low denotes levels 0–2, medium 3–4 and high 5–6) and employment status.

### Macro-level Variables

To account for the multidimensionality of gender regimes, we used two macro-level indicators to capture normative/attitudinal and institutional aspects. These are our key explanatory variables for analysing cross-country differences in terms of the effect of religiosity on fertility. First, we constructed a Gender Attitudes Index from four items included in the GGS regarding attitudes towards gender roles and relationships, based on the approach used by Aassve et al. (2015). For each of the following four items, respondents were asked about the extent to which they agreed or disagreed with the following statements (on a 5-point scale from “strongly agree” to “strongly disagree”):

- In a couple, it is better for the man to be older than the woman
- If a woman earns more than her partner, it is not good for the relationship
- On the whole, men make better political leaders than women do
- When jobs are scarce, men should have more right to a job than women

We recoded the answers to each question in a scale from 1 (least traditional gender attitude) to 5 (most traditional gender attitude). The average score for each respondent was then calculated. These data were then used to calculate the country average. Different from our individual-level variables, we used all respondents in the dataset (i.e., aged 18 and over^2^) to calculate country averages. This was necessary because we are interested in the gender-attitude environment, which is rooted in norms, attitudes and cultural habits, all of which can be formed and upheld by people of all ages. Factor analysis confirms that all four items belong to one factor. Across the whole sample, Cronbach’s alpha amounted to 0.70, deemed an acceptably high value, with some variation between countries. (It ranged from 0.82 in Sweden to 0.46 in Russia.)

In order to incorporate the institutional aspect of the gender regime, it would have been appropriate to construct an index that represents, for example, institutional support for the dual-earner model. Unfortunately, indices of that kind providing data on all countries of interest were not available. Most such indices are only available for EU countries (e.g. Billingsley and Ferrarini 2014; Matysiak and Weziak-Bialowolska 2016). For this reason, we used the Economic Participation and Opportunity sub-index (EPO-Index) as an alternative, which is part of the Gender Gap Index developed by the World Economic Forum (2008). This sub-index measures the extent to which women are able to participate in the economy. It ranges from 0 (least possible participation and opportunities) to 1 (highest possible participation and opportunities).

We acknowledge that the Gender Attitudes Index and the EPO-Index might not be a perfect means of representing the gender regime. Therefore, we also conducted robustness checks using a wide array of other indicators related to gender regime. These are described in detail in the “Robustness check” section.

### Methods

For modelling the micro–macro-linkages in our paper, we followed the two-step approach described by Bryan and Jenkins (2016) involving meta-analysis and meta-regression. This approach has some advantages compared to other approaches like multilevel modelling, which have been often used for similar research questions. Bryan and Jenkins (2016) argued that for multilevel logistic regression, at least 30 clusters are necessary to allow for reliable estimates to be made. For 15 clusters, country-level variance is already biased downwards by 10% and may be even more biased in our case of 12 countries. The two-step approach does not only show unbiased estimates, but also provides a graphical representation of country-level variations which give further clues about them.

In the first step of this approach, we ran logistic models for each country separately using the individual-level variables. The coefficients and standard errors expressed in terms of odds ratio or logit coefficients of the variable of interest as required in the meta-regression cannot be used to compare the strength of effect across different countries (Mood 2009). Therefore, we used average marginal effects, measured in percentage points. They show the average difference in the probability that the event in question takes place for each possible value of the categorical variable, in comparison with the reference category.

These results were used to perform a meta-analysis using the “metan” command in Stata (Harris et al. 2008). Because religiosity in our study is a categorical variable with three possible values, there are two indicators of the effect strength in each model: the first describes the effect of attending religious services less than monthly compared to never, and the second describes the effect of attending monthly or more compared to never. Meta-analysis provides, among other things, estimates of heterogeneity (in other words, the variation between studies, in this case countries, rather than within-study variance), and the *I*^2^ statistic showing the variation in size of effect attributable to heterogeneity across countries (Higgins and Thompson 2002).

In the second step, we performed the actual meta-regression at the macro-level, using the “metareg” command (Harbord and Higgins 2008). Theoretically, a simple linear regression would be possible using our gender regime indices as the independent variable, and the strengths of effect from the meta-analysis as the dependent variable. Meta-regression nevertheless has the advantage that it accounts for the standard errors of the effects of religiosity, as well as for the number of observations in each country.

Our macro-level hypotheses were framed as directional hypotheses, i.e., we hypothesized that each effect has a defined direction. In the analyses, we therefore applied one-sided t tests.

The procedure described above was repeated for both sexes (male and female), both time horizons (short- and long-term intentions) and both macro-level variables (Gender Attitudes Index and EPO-Index), leading to a total of 8 models with two meta-regressions per model: one for the effect of medium religiosity and the other for the effect of high religiosity, compared to our reference category of low religiosity.

## Results

### Descriptive Results

The distributions of our variables according to country are shown in the Appendix in Table 3 for men and in Table 4 for women. The percentage of respondents intending to have a(nother) child over the next three years varies between countries. Georgia is the country with the highest short-term intentions (men: 43% and women: 34%), whereas German respondents show the lowest short-term intentions (around 21% for both women and men). For long-term intentions, the pattern is slightly different, with Austrian men and Swedish women showing the highest intentions to have a(nother) child at any time (63% and 62%, respectively). Norwegian men and Romanian women have the lowest intentions (39% in both cases). Overall, it is also important to note that among men, cross-country differences are more pronounced than among women.

In terms of religious practices and denominations, there are notable cross-country differences as well. Respondents from France and Czechia are the least frequent attenders of religious services, with a vast majority stating that they never attend them. In France, however, most respondents still consider themselves Christian, while Czechia is the only country in our sample where the majority of respondents do not belong to any religion. Polish, Romanian and Georgian respondents, on the other hand, are the most likely to attend services monthly or more. They are also the least likely to belong to no religion at all. Overall, women are more likely to belong to a religious denomination and attend religious services more frequently than men. These gender differences in religiosity tend to be more pronounced among Eastern European countries, especially so in Bulgaria, Russia, Georgia and Romania.

The Gender Attitudes Index and the EPO-Index are macro-indicators, and therefore the same for both genders. Expressed in the Gender Attitudes Index, Sweden and Norway can be considered the least traditional, while Georgia, followed by Russia, occupies the other end of the spectrum. In terms of the EPO-Index, there are some marked differences. Again, Sweden and Norway show the highest index values; Austria and Poland show the lowest values. Russia and Latvia are examples of countries that score high in terms of the EPO-Index, despite scoring lower on the Gender Attitudes Index.

### Meta-Analysis of the Models

In the meta-analysis, we systematically analysed the strength of the effect of religiosity on the likelihood of the intention to have a(nother) child, as well as the extent to which these effects vary by country. The forest plots (Figs.  5 and 6 in the Appendix) depict the average marginal effect on the probability of intending to have a(nother) child for each country separately and at the bottom—an overall average value for all countries. The values are expressed in percentage terms and show how much more likely a respondent of medium or high religiosity is to intend to have children compared to the reference category (low religiosity). They also show the confidence interval associated with each value. The dotted vertical line shows the overall effect among all countries combined with the confidence interval, indicated by the diamond at the bottom of each diagram.

The overall effects are positive in all cases, meaning that the probability of respondents intending to have a(nother) child both in the short and long term is highest for those who attend religious services at least once a month. Those attending less than monthly are less likely to plan to have a child than the highly religious but are more likely to do so in comparison with those who never attend. The overall effect varies only very slightly according to the timeframe of the intentions. For men, the effects are slightly stronger than for women.

However, significant positive effects of religiosity on fertility intentions are not seen in all countries. The number of countries with a positive effect also varies by timeframe, religiosity level compared and gender. Highly religious men are significantly more likely to intend to have a child in the long-term perspective than their less religious counterparts in six countries: Bulgaria, Russia, France, Romania, Poland and Czechia. It needs to be noted, however, that in Russia, France and Czechia, highly religious men constitute a rather small share of the respondents. For women, significant effects were found in seven countries: Bulgaria, Russia, Germany, Norway, Austria, Poland and Czechia. In contrast, there are only three countries with significant positive effects for medium religious respondents and short-term intentions (Bulgaria and Russia for both men and women, Georgia for men, Norway for women). For men, Bulgaria and Russia show positive effects in all models, while in Germany, Austria, Norway and Sweden, there are no significant effects in any of the models. In the case of women, only Russia and Norway show significantly positive effects in all models, while there are no significant effects in any of the models in Georgia, France, Romania, Lithuania and Sweden.

Generally, the country differences in the effects of religiosity on fertility intentions as revealed by the meta-analysis are small. The I-squared values are generally higher for high vs. medium religiosity and for long-term vs. short-term intentions, indicating greater cross-country variation in the effects in those situations. For both men and women, the most pronounced differences occur for the model for high religiosity coupled with long-term intentions (I-squared of over 50%).

### Meta-Regression Models

**Fig. 1:**
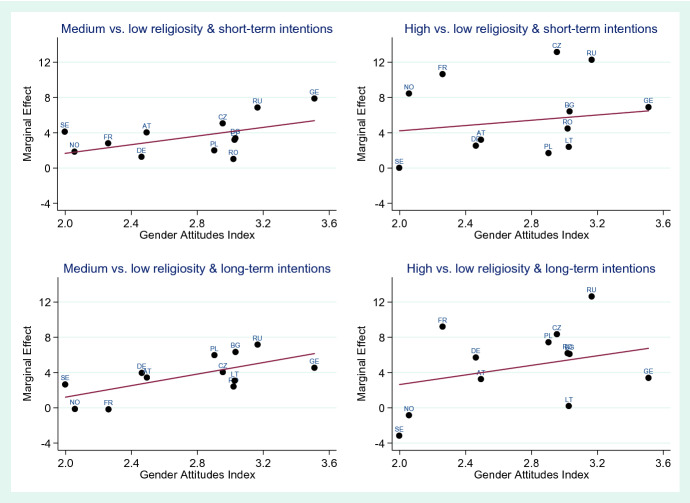
Meta-regression results of the association of the Gender Attitudes Index with the effect strength of religiosity on fertility intentions for short-term and long-term fertility intentions, country-specific effects expressed as average marginal effects in percentage points, men

**Fig. 2:**
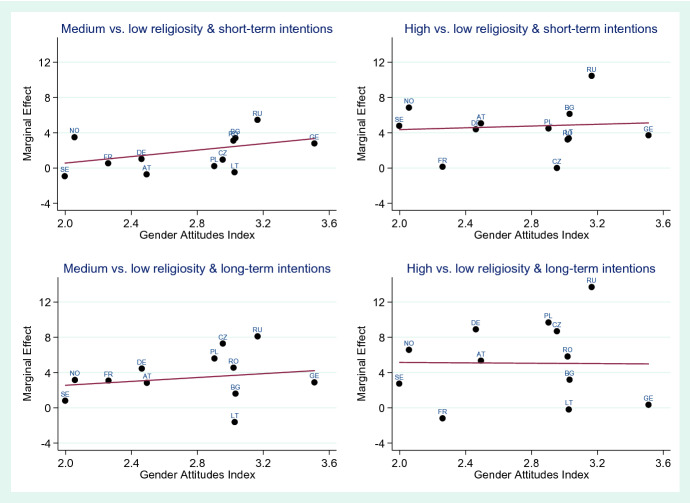
Meta-regression results of the association of the Gender Attitudes Index with the effect strength of religiosity on fertility intentions for short-term and long-term fertility intentions, country-specific effects expressed as average marginal effects in percentage points, women

Figures 1 and 2 show the results of the meta-regressions in graphical terms, using marginal effects as the dependent variable and the Gender Attitudes Index as the independent variable. Table 5 in the Appendix shows the corresponding coefficients and *p* values for all meta-regressions. For men, the regression line has a positive gradient, suggesting that in more traditional countries, religiosity has a stronger effect on fertility intentions than in more egalitarian countries. However, only in the case of long-term intentions and medium religiosity is this association significant. In the case of women, the gradients of the regression line are positive in all cases except for high religiosity and short-term intentions. None of these associations are statistically significant, however.

**Fig. 3:**
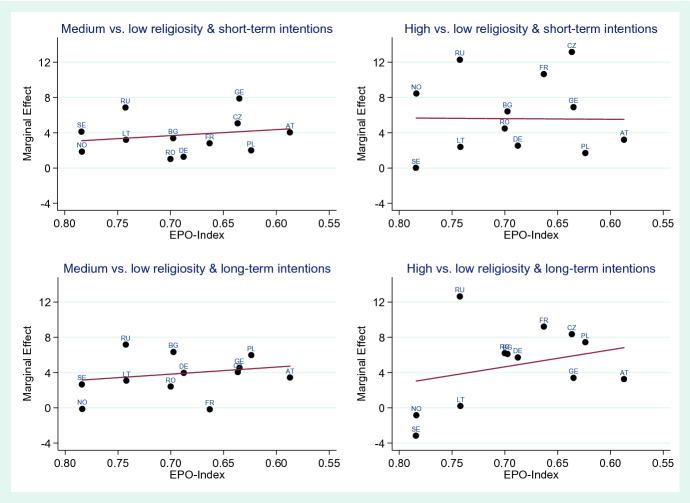
Meta-regression results of the association of the EPO-Index with the effect strength of religiosity on fertility intentions for short-term and long-term fertility intentions, country-specific effects expressed as average marginal effects in percentage points, men

**Fig. 4:**
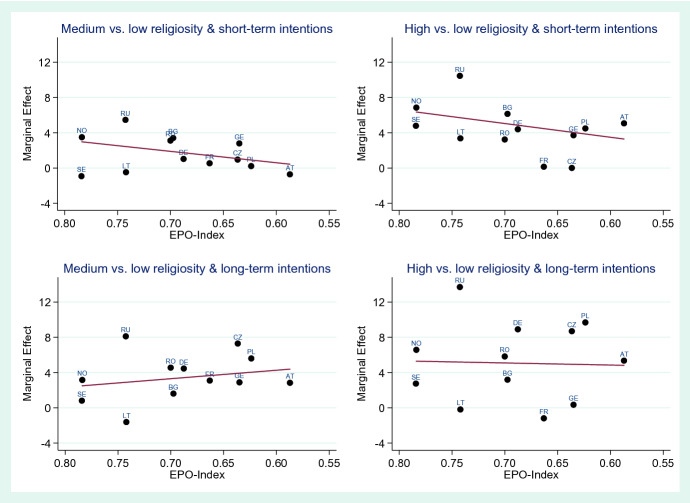
Meta-regression results of the association of the EPO-Index with the effect strength of religiosity on fertility intentions for short-term and long-term fertility intentions, country-specific effects expressed as average marginal effects in percentage points, women

In Figs. 3 and 4, we show the same analyses repeated for the EPO-Index (on economic opportunities and participation for women). The index on the x-axis is shown from high-to-low values from left to right, such that the more traditional countries lie on the right-hand side in each graph. Compared to the results using the Gender Attitudes Index, the pattern is less clear. While in some cases, the line shows a higher strength of effect of religiosity on fertility intentions in more traditional countries, none of these associations are statistically significant. These results thus overall imply that the effect strength of religiosity on fertility intentions in a country is not dependent on the amount of economic opportunities or on the level of women’s participation in the economy.

**Fig. 5:**
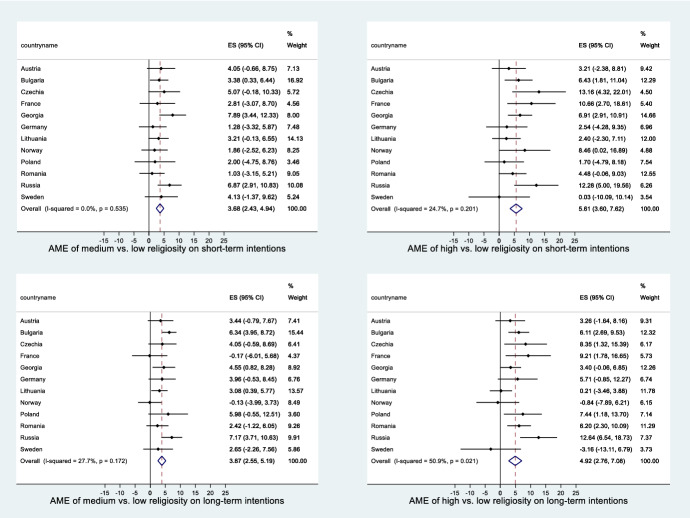
Country-specific effects and the overall effect of high and medium versus low religiosity on short- and long-term fertility intentions, men (meta-analysis). The effect strengths are expressed as average marginal effects (AME) in percentage points

**Fig. 6:**
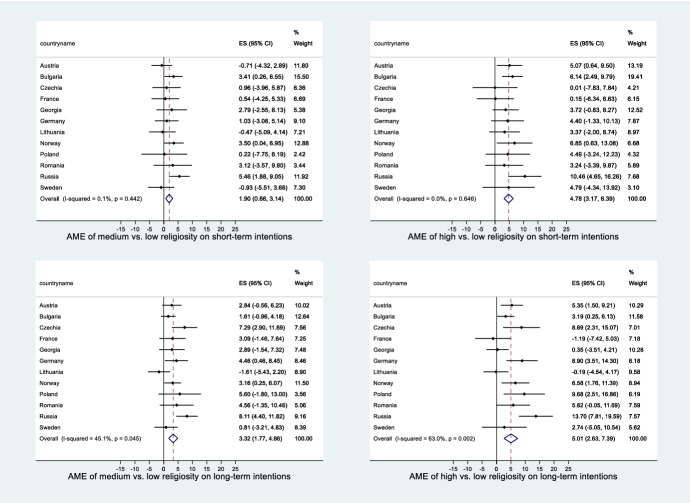
Country-specific effects and the overall effect of high and medium versus low religiosity on short- and long-term fertility intentions, women (meta-analysis). The effect strengths are expressed as average marginal effects (AME) in percentage points

### Robustness Checks

To account for possible limitations of the macro-level indicators of gender regime used in our study, we decided to conduct robustness checks using different indicators on gender equality. Similar to the Gender Attitudes Index in our models, we constructed an index using 8 items on gender equality from the 4th wave of the European Values Study. Additionally, we tested the influence of other indices on gender equality: the Gender Development Index and Gender Empowerment Measure (United Nations 2007), the Gender Inequality Index (United Nations 2010), the Gender Equity Index (Social Watch 2008), the Gender Gap Index (World Economic Forum 2008) and the Index of conditions for work and family reconciliation (ICWFR) developed by Matysiak and Weziak-Bialowolska (2016). In order to make the indices more comparable, standardized scores were used and some indices were reversed in order to ensure that for all of them, higher values are associated with a more traditional gender regime Table 1.

**Table 1:** Results of the meta-analysis of the effect of religiosity on short-term and long-term fertility intentions in 12 countries

		Medium versus low religiosity	High versus low religiosity
Men	Short term	BG, GE, RU	BG, CZ, FR, GE, NO, RU
	Long term	BG, GE, LT, RU	BG, CZ, FR, PL, RO, RU
Women	Short term	BG, NO, RU	AT, BG, NO, RU
	Long term	CZ, DE, NO, RU	AT, BG, CZ, DE, NO, PL, RU

Shown here are those countries with significant positive effects of religiosity on fertility intentions

In Table 2, each coefficient and its* p* value represent a meta-regression. A positive coefficient means that the more traditional a country is on the respective gender index, the stronger the effect of religiosity. The p values show whether that coefficient is statistically significant or not. These alternative indicators do not add a great deal to the overall picture, however. Almost all coefficients are insignificant, and the few significant coefficients do not show a consistent pattern. The gender indices are mostly relevant in determining the strength of effect of religiosity on fertility intentions in the case of the long-term intentions of men.

**Table 2:** Overview of the results of separate meta-regressions of the association of various gender-related indices with the effect strength of religiosity on fertility intentions (robustness checks). Shown here are standardized meta-regression coefficients and their *p* values

		Number of countries	Short term				Long term			
			Medium versus low religiosity		High versus low religiosity		Medium versus low religiosity		High versus low religiosity	
			Std. coeff	*p*	Std. coeff	*p*	Std. coeff	*p*	Std. coeff	*p*
Men	Gender Attitudes Index	12	1.17	0.066	0.71	0.284	1.55	0.021	1.29	0.172
	EPO-Index	12	0.44	0.282	− 0.05	0.515	0.51	0.265	1.23	0.183
	EVS-Index	12	0.66	0.178	− 0.89	0.749	0.10	0.095	0.91	0.260
	Gender Development Index 2008	11	0.51	0.241	0.58	0.340	0.17	0.011	2.01	0.077
	Gender Empowerment Measure 2008	12	1.14	0.067	1.17	0.152	1.34	0.036	1.89	0.072
	Gender Equity Index 2008	12	0.99	0.108	1.12	0.183	0.84	0.148	1.73	0.110
	Gender Gap Index 2008	12	0.85	0.134	0.80	0.285	1.34	0.042	2.37	0.047
	Gender Inequality Index 2008	12	1.20	0.059	0.78	0.231	1.09	0.076	0.77	0.271
	ICWFR, Matysiak 2016	10	− 0.12	0.557	− 0.69	0.670	0.86	0.180	2.26	0.081
Women	Gender Attitudes Index	12	0.87	0.125	0.24	0.399	0.52	0.279	− 0.05	0.514
	EPO-Index	12	− 0.82	0.877	− 1.00	0.853	0.61	0.244	− 0.15	0.542
	EVS-Index	12	− 0.13	0.566	− 0.28	0.611	0.29	0.371	0.17	0.452
	Gender Development Index 2008	11	1.27	0.042	1.05	0.139	0.68	0.245	1.11	0.229
	Gender Empowerment Measure 2008	12	1.06	0.078	0.12	0.446	0.86	0.165	− 0.05	0.514
	Gender Equity Index 2008	12	0.21	0.397	− 0.95	0.823	0.77	0.195	− 0.69	0.679
	Gender Gap Index 2008	12	0.29	0.354	− 0.52	0.692	0.88	0.156	0.17	0.455
	Gender Inequality Index 2008	12	1.18	0.072	0.21	0.405	0.43	0.322	− 0.62	0.671
	ICWFR, Matysiak 2016	10	− 0.40	0.690	− 0.99	0.777	0.85	0.169	1.14	0.211

## Discussion and Conclusions

The aim of our study was to add evidence to the ongoing discussion on the relationship between religiosity and fertility. Specifically, we attempted to explain cross-country differences in the influence of religiosity on fertility intentions, as found in a previous study (Philipov and Berghammer 2007). Based on our theoretical considerations and on previous research that suggested that relationship between religiosity and fertility differs in more and less traditional countries (Guetto et al. 2015), we posited that the gender regime might help to explain these differences.

Our first aim was to confirm an overall association between childbearing plans and religiosity. We hypothesized (hypothesis H1a) that frequent attendance at religious services is positively associated with these plans. We found this to be the case, as far as the overall effect across countries is concerned. Regardless of gender, and for both short- and long-term fertility intentions, compared to the non-religious, people of medium religiosity are more likely to intend to have a(nother) child, and highly religious people are even more likely to have such intentions. These findings were only partially confirmed when considering the effects for each country separately, however. The overall positive effect is not mirrored in all the countries included in our study, corroborating the findings of previous research (Philipov and Berghammer 2007).

In our analyses, we included both short- and long-term fertility intentions and hypothesized that religiosity has more of a bearing for the latter (H1b). The meta-analysis provided no support for this hypothesis: there are no notable differences between the average sizes of the effect of attendance at religious services on short-term and long-term intentions. There is some support for H1b, if one considers the number of countries in which statistically significant positive effects can be found. This number is slightly higher in the case of long-term than short-term intentions.

Because our findings confirm a large variation in the degree to which religiosity influences childbearing intentions across countries, we made use of meta-regression to evaluate whether this effect is stronger in countries with more traditional gender regimes (hypothesis H2). We tested our hypothesis using two different indices: the Gender Attitudes Index, representing societal norms and ideals in terms of gender roles, and the EPO-Index, representing the institutional dimension of the gender regime. Moreover, we conducted robustness checks using an array of other indicators representing various aspects of the gender regime.

Our analyses revealed that for the long-term childbearing intentions of men, the effect of religiosity is indeed stronger in more traditional countries, but this association was only found when men who never attend religious services were compared with those with medium levels of attendance, and only when the Gender Attitudes Index was considered. No significant association was found for the EPO-Index. As explained below, this may be related to the nature of the EPO-Index itself. The robustness checks largely confirmed this conclusion: several indices are able to explain differences only in the strength of effect of religiosity for the long-term intentions of men.

Our finding that in more traditional gender regimes, the effect of religiosity on fertility intentions is stronger for men but not for women calls for some explanation. First, we need to consider that with the second phase of the Gender Revolution on the way (F. Goldscheider et al. 2015), some religious institutions have adjusted their doctrines and have increasingly begun to promote gender equality and the involvement of men in the household and private sphere. This has become evident in the Church of Sweden (Church of Sweden 2012; F. Goldscheider et al. 2014) and the Evangelical Church of Germany (Rat der Evangelischen Kirche in Deutschland 2013). Consequently, in more progressive countries, religious men may have scaled down their fertility intentions, because a higher level of gender equality places more responsibilities on them in the household and childrearing spheres. This effect is not visible in more traditional settings.

For women, the adjustment of religious doctrines towards higher gender equality—observed in egalitarian gender regimes—may have a different effect. A more permissive stance by religious institutions towards women seeking to combine career and children will encourage highly religious women to join the labour market. Therefore, highly religious women could, similarly to the less religious women, profit from family policies associated with more egalitarian gender regimes. It follows that both the highly and the less religious women are more likely to intend to have a child in egalitarian countries compared to traditional countries. Therefore, the positive effect of religiosity on fertility intentions remains similar regardless of the gender regime in the case of women, and especially so when the EPO-Index on economic opportunities is considered.

Additionally, it should be considered that parenthood may carry different meanings for men and women. Women are generally more inclined to intend to have children, while men are more likely to intend to stay childless (Miettinen and Szalma 2014). With parenthood being a more universal ideal for women than for men, there might be more variance in men’s fertility intentions, which can be explained by, among other things, religiosity. Contrary to the effect of the adoption of gender equality by religions as described above, this effect largely applies in more traditional countries.

Furthermore, the differences in the extent to which welfare states support families might be important. In traditional, familialist countries such as Poland, such support is generally rather limited and families are expected to care for themselves (Javornik 2014). Men in these countries are expected to provide the financial means to support their families. In case of unemployment or other economic hardship, for example, non-religious men might limit their fertility intentions significantly, because they are no longer able to support further children. More religious men in that situation, on the other hand, might maintain their intention to have a child, because their faith could give them support in coping with economic hardship (Philipov 2011). Overall, this may lead to a bigger difference in fertility intentions between more and less religious men in more traditional countries.

More egalitarian gender regimes, on the other hand, often have extensive welfare states that provide support during times of economic hardship. Additionally, men are not expected to be the sole breadwinners, and they might therefore be less likely to give up their fertility intentions in these situations. Here, the gap between more and less religious men is probably much smaller.

In summary, our research showed that the gender regime is able to explain only a small fraction of the variation in the effect of religiosity on fertility intentions, and only as far as men’s intentions are concerned. This study can therefore be considered a starting point and could be expanded along several lines of possible enquiry.

One point concerns the measurement of religiosity, in our case the frequency of attending religious services. One criticism that has been made by some researchers regarding this indicator is that the meaning of attending religious services could vary between religious denominations. Catholicism, for example, puts a higher priority on churchgoing than other Christian denominations (Voas and Doebler 2011). A high frequency of attending religious services may indicate a very high level of religiosity in a context of a low attendance frequency. In such context, the highly religious constitute a very selected group. In our analyses, this turned out to be the case for men from Czechia, France and Russia. In a context of high attendance on the other hand, the attendance frequency may not be able to discriminate well enough between the highly religious and those that mostly attend to follow local traditions. This was found in the context of 1970s Spain (Adserà 2006a), and it applies to Poland in our research. In such contexts, the less religious are a potentially selected group. It would have been appropriate for these contexts to further differentiate those highly religious people into more nuanced categories. For example, it could be suggested to create a category of “people with a very high religiosity”, who attend religious services more often than weekly. This approach would not have worked in less religious contexts, however, as the number of cases in this very highly religious group would have been too small to be analysed. Notably, in our results we did not find differences in the likelihood of getting significant effects in different religious contexts, suggesting that the problem of selected groups did not affect our results in any significant way and supporting our choice of grouping the attendance frequency into the three categories. Moreover, as we focused on Christian denominations most of all, the frequency of attending religious services is likely to be interpreted in a comparable way across countries and carries a similar meaning among our respondents (Brenner 2016). Nevertheless, other measures of religiosity could be tested in future studies to corroborate our findings.

Similarly, future research could examine other stages of the fertility decision-making process besides fertility intentions, such as fertility ideals or behaviour. Due to the persistence of the two-child “ideal” in Europe, parity-specific effects might also be expected (Sobotka and Beaujouan 2014), meaning that the relationship between religiosity and fertility and the influence of the gender regime could be parity-specific. It might also be possible that other characteristics interact with gender regime, such as education or partnership status. In our analyses, we controlled for these individual-level variables, but their moderating effect was not tested.

Our study includes 12 European countries and focuses mostly on the impact of Christian religiosity on fertility intentions, and even for these (mostly) Christian countries, the study could be further developed. In our paper, the traditional side of the Gender regime is mostly represented by post-communist countries in Central and Eastern Europe, and due to their historical heritage, their behaviour in our models may be different from otherwise similarly traditional countries in Southern Europe, such as Italy, Greece or Spain. For a better understanding of the influence of different gender regimes, it would be beneficial to add these countries in future studies. Furthermore, highly religious adherents of other religions might react differently to the gender regime from Christians, meaning that one future avenue of research would be to examine the universality of our findings across other religious and cultural settings.

Technically, a sample of 12 countries is sufficiently large to allow meta-regression analyses (Valentine et al. 2010), but using a greater number of countries could also improve the reliability of the coefficients. Similarly, meta-regression is sensitive to the confidence intervals of the sizes of effects within each country, which are still rather high in our case of fertility intentions.

Finally, this research could also be expanded by taking another approach to measuring the gender regime. Despite making use of many different indices, we relied on one-dimensional measures, while more compound measures could be more appropriate to capture the complexity of gender regime. Beyond using a different approach on gender regime, it might also be possible that other dimensions of macro-level context influence the relationship between religiosity and fertility across countries.

## Appendix

See Tables

**Table 3:** Descriptive analysis of the data by country including (a) average values of the Gender Attitudes Index and the EPO-Index and (b) the distribution of the male sample by characteristics

		Austria	Bulgaria	Czechia	France	Georgia	Germany	Lithuania	Norway	Poland	Romania	Russia	Sweden
Gender Attitudes Index		2.493	3.031	2.954	2.260	3.510	2.462	3.026	2.056	2.903	3.019	3.165	1.997
EPO-Index		0.587	0.698	0.637	0.663	0.635	0.688	0.742	0.784	0.624	0.700	0.743	0.784
		%	%	%	%	%	%	%	%	%	%	%	%
Attendance frequency	Never	44.5	40.8	77.6	82.3	36.2	38.1	30.7	57.9	5.1	12.5	59.9	59.0
	Less than monthly	33.2	46.5	15.7	10.5	24.8	47.1	54.3	35.6	26.3	55.0	33.3	34.6
	Monthly and more often	22.3	12.7	6.7	7.2	39.0	14.7	15.1	6.5	68.6	32.4	6.8	6.3
Short-term intentions (yes)		28.6	30.9	28.6	24.6	29.4	42.8	20.6	24.2	24.9	30.1	28.9	27.7
Long-term intentions (yes)		51.5	62.6	51.5	50.1	46.9	57.3	41.6	55.1	38.8	46.0	47.1	56.0
Religion	Christian	74.5	75.1	22.5	69.4	86.1	64.9	89.1	81.0	98.9	99.3	64.5	56.2
	Other Religion	8.1	13.2	2.1	7.3	12.7	6.8	0.9	5.4	0.9	0.4	8.0	5.6
	Unaffiliated	17.4	11.6	75.0	15.8	1.2	27.8	9.5	13.3	0.0	0.2	27.5	35.1
	Missing	0.0	0.1	0.4	7.5	0.0	0.6	0.5	0.3	0.2	0.1	0.0	3.1
Age category	18–24	23.7	22.4	19.9	23.4	25.4	15.9	27.6	10.4	14.0	16.0	25.7	21.7
	25–29	17.2	15.2	17.5	14.1	14.8	12.9	14.9	11.1	15.0	13.2	16.1	13.7
	30–34	16.3	17.2	16.7	15.3	15.4	16.4	16.0	17.2	20.3	17.2	14.4	15.6
	35–39	18.8	17.3	16.1	18.8	15.5	18.9	14.7	24.4	20.7	22.7	14.0	18.1
	40–49	24.0	28.0	29.7	28.4	28.9	35.8	26.7	36.9	29.9	31.0	29.8	31.0
Number of biological children	0	57.9	44.8	49.2	48.0	44.8	47.8	45.5	28.4	29.4	40.1	38.1	54.1
	1	16.2	22.5	17.3	16.8	13.8	22.2	24.5	15.3	26.3	27.9	30.5	14.1
	2	17.9	27.4	26.5	23.4	29.2	22.4	24.2	34.6	30.6	24.4	25.0	24.2
	3 and more	8.0	5.4	7.0	11.8	12.3	7.5	5.8	21.6	13.8	7.5	6.3	7.7
Young child (below 10) (yes)		26.7	29.8	26.7	25.8	38.2	33.2	31.3	28.6	52.6	47.8	30.9	30.8
Partnership status	Coresiding	47.4	56.0	57.7	56.9	55.7	63.0	59.3	83.3	80.6	65.7	64.0	59.5
	Not living with partner	52.5	43.8	42.1	43.1	44.3	36.2	40.7	16.5	19.4	34.3	35.6	40.5
	Missing	0.1	0.3	0.2	0.0	0.0	0.7	0.0	0.2	0.0	0.0	0.3	0.0
Education	Low	11.0	23.5	17.8	20.5	7.3	16.8	14.4	19.7	9.1	21.8	7.3	11.6
	Medium	72.2	62.8	64.4	51.7	64.4	59.5	64.9	48.5	66.5	66.9	55.6	55.0
	High	16.8	13.7	16.1	27.7	28.2	23.7	20.7	30.2	24.2	11.4	30.9	32.9
	Missing	0.0	0.1	1.8	0.0	0.0	0.0	0.0	1.6	0.3	0.0	6.2	0.4
Employment status	In paid work	82.5	65.5	78.8	73.3	60.4	75.4	74.1	88.4	81.9	80.9	74.4	77.1
	Not in paid work	17.5	34.5	21.2	26.7	39.6	24.6	25.9	11.6	18.1	19.1	25.6	22.9
N		1990	2940	2005	2166	2420	1654	2487	1220	2929	2885	2230	906

^3^,

**Table 4:** Descriptive analysis of the data by country including (a) average values of the Gender Attitudes Index and the EPO-Index and (b) the distribution of the female sample by characteristics

		Austria	Bulgaria	Czechia	France	Georgia	Germany	Lithuania	Norway	Poland	Romania	Russia	Sweden
Gender Attitudes Index		2.493	3.031	2.954	2.260	3.510	2.462	3.026	2.056	2.903	3.019	3.165	1.997
EPO-Index		0.587	0.698	0.637	0.663	0.635	0.688	0.742	0.784	0.624	0.700	0.743	0.784
		%	%	%	%	%	%	%	%	%	%	%	%
Attendance frequency	Never	38.9	24.8	72.8	77.8	27.6	33.2	17.7	57.2	2.2	5.7	41.8	54.1
	Less than monthly	37.5	50.8	18.7	14.1	20.5	50.4	58.5	35.1	22.4	40.5	48.4	39.3
	Monthly and more often	23.6	24.4	8.5	8.0	51.9	16.5	23.8	7.7	75.4	53.9	9.8	6.7
Short-term intentions (yes)		24.0	28.8	24.0	27.3	32.7	34.2	21.3	24.6	26.8	28.4	26.1	21.3
Long-term intentions (yes)		40.8	54.9	40.8	48.1	50.0	48.0	42.0	53.7	51.7	47.3	39.0	44.3
Religion	Christian	78.9	81.0	24.6	72.8	87.7	66.4	93.0	80.6	99.0	99.6	76.7	65.7
	Other Religion	7.3	12.4	1.4	7.1	11.4	7.9	0.5	6.6	0.8	0.3	6.3	5.2
	Unaffiliated	13.6	6.4	73.7	14.5	0.9	25.1	6.1	12.7	0.0	0.1	17.0	26.2
	Missing	0.1	0.2	0.3	5.6	0.0	0.6	0.5	0.1	0.2	0.0	0.0	2.8
Age category	18–24	24.3	21.1	23.5	25.8	24.6	22.4	31.6	26.2	21.0	15.4	18.5	27.8
	25–29	18.3	17.3	20.8	16.0	18.6	16.0	15.8	17.2	19.3	17.7	18.2	18.7
	30–34	17.3	20.2	21.4	17.5	17.5	18.1	19.7	17.8	21.9	22.2	18.7	16.9
	35–39	19.7	20.5	17.8	21.4	19.8	22.3	16.5	22.7	21.8	28.9	17.8	18.5
	40–44	20.3	20.8	16.5	19.2	19.5	21.2	16.4	16.0	16.0	15.9	26.8	18.1
Number of biological children	0	49.4	29.6	36.2	41.9	35.1	40.3	39.5	46.9	30.2	27.0	12.5	52.5
	1	20.5	28.4	22.9	19.5	16.7	24.9	28.0	14.6	27.1	34.6	45.7	13.6
	2	22.3	36.1	32.0	23.9	33.9	24.6	26.7	25.4	30.8	29.6	34.1	24.8
	3 and more	7.8	5.9	8.8	14.7	14.2	10.2	5.8	13.1	11.9	8.8	7.7	9.0
Young child (below 10) (yes)		33.0	33.1	33.0	35.4	43.6	37.7	39.5	34.3	40.1	48.6	39.8	46.8
Partnership status	Coresiding	54.5	66.1	64.1	62.4	59.4	64.3	52.6	58.5	67.2	74.6	72.2	62.8
	Not living with partner	45.5	33.8	35.6	37.6	40.6	35.2	47.4	41.3	32.8	25.4	27.6	37.2
	Missing	0.0	0.1	0.2	0.0	0.0	0.5	0.0	0.2	0.0	0.0	0.2	0.0
Education	Low	14.2	19.6	15.8	15.6	8.7	23.8	11.4	20.7	9.5	27.1	4.7	14.2
	Medium	66.9	53.7	70.1	46.1	61.5	57.4	62.6	36.6	58.9	60.5	45.3	45.9
	High	18.8	26.7	12.7	38.3	29.8	18.8	26.0	39.8	31.1	12.4	44.2	39.5
	Missing	0.0	0.1	1.3	0.0	0.0	0.0	0.0	2.8	0.5	0.0	5.9	0.4
Employment status	In paid work	72.8	59.6	56.6	59.9	32.3	55.3	59.8	67.0	55.1	62.4	63.3	63.6
	Not in paid work	27.2	40.4	43.4	40.1	67.7	44.7	40.2	33.0	44.9	37.6	36.7	36.4
N		2156	3093	1923	2255	2481	1945	2579	2094	4269	2138	2465	1199

^4^,

**Table 5:** Coefficients and *P* values of the meta-regressions of the association of the Gender Attitudes Index and the EPO-Index with the effect strength of religiosity on short- and long-term fertility intentions

		Men				Women			
		Short term		Long term		Short term		Long term	
		Medium versus low religiosity	High versus low religiosity	Medium versus low religiosity	High versus low religiosity	Medium versus low religiosity	High versus low religiosity	Medium versus low religiosity	High versus low religiosity
Gender Attitudes Index	Coef	2.45	1.50	3.27	2.72	1.84	0.50	1.10	1.10
	*p*	0.066	0.284	0.021	0.172	0.125	0.399	0.279	0.279
	Intercept	− 3.24	1.23	− 5.33	− 2.80	− 3.12	3.36	0.36	0.36
	*p*	0.768	0.436	0.894	0.636	0.767	0.275	0.472	0.472
EPO-Index	Coef	− 6.84	0.76	− 8.01	− 19.28	12.90	15.57	− 9.60	− 0.11
	*p*	0.718	0.485	0.736	0.818	0.124	0.147	0.244	0.514
	Intercept	8.46	5.07	9.43	18.14	− 7.14	− 5.85	10.02	5.36
	*p*	0.159	0.357	0.149	0.112	0.823	0.722	0.154	0.267

^5^ and Figs 5, 6.

## Footnotes

1 The following countries were excluded for the following reasons: for Australia, Belgium, Estonia, Hungary and Japan, no data on religiosity were available. Data on Belarus have not yet been published. For Italy and the Netherlands, the variables necessary for the construction of the Gender Attitudes Index were not available.

2 In Austria, the GGS sample only consisted of respondents aged 18–45, while in all other countries in our study, the age range is 18–80. Therefore, the Gender Attitudes Index for Austria might be slightly biased.
